# Tree shoot bending generates hydraulic pressure pulses: a new long-distance signal?

**DOI:** 10.1093/jxb/eru045

**Published:** 2014-02-20

**Authors:** Rosana Lopez, Eric Badel, Sebastien Peraudeau, Nathalie Leblanc-Fournier, François Beaujard, Jean-Louis Julien, Hervé Cochard, Bruno Moulia

**Affiliations:** ^1^Anatomía, Fisiología y Genética vegetal, ETSI Montes, Universidad Politécnica de Madrid, Spain; ^2^INRA, UMR547 PIAF, F-63100 Clermont-Ferrand, France; ^3^Clermont Université, Université Blaise Pascal, UMR547 PIAF, F-63000 Clermont-Ferrand, France

**Keywords:** Bending, conductivity, hydraulic, mechanosensing, poroelasticity, pressure, signalling, strain, trees, wood, water.

## Abstract

When tree stems are mechanically stimulated, a rapid long-distance signal is induced that slows down primary growth. An investigation was carried out to determine whether the signal might be borne by a mechanically induced pressure pulse in the xylem. Coupling xylem flow meters and pressure sensors with a mechanical testing device, the hydraulic effects of mechanical deformation of tree stem and branches were measured. Organs of several tree species were studied, including gymnosperms and angiosperms with different wood densities and anatomies. Bending had a negligible effect on xylem conductivity, even when deformations were sustained or were larger than would be encountered in nature. It was found that bending caused transient variation in the hydraulic pressure within the xylem of branch segments. This local transient increase in pressure in the xylem was rapidly propagated along the vascular system *in planta* to the upper and lower regions of the stem. It was shown that this hydraulic pulse originates from the apoplast. Water that was mobilized in the hydraulic pulses came from the saturated porous material of the conduits and their walls, suggesting that the poroelastic behaviour of xylem might be a key factor. Although likely to be a generic mechanical response, quantitative differences in the hydraulic pulse were found in different species, possibly related to differences in xylem anatomy. Importantly the hydraulic pulse was proportional to the strained volume, similar to known thigmomorphogenetic responses. It is hypothesized that the hydraulic pulse may be the signal that rapidly transmits mechanobiological information to leaves, roots, and apices.

## Summary

Bending of trees causes a transient hydraulic overpressure signal that propagates rapidly along the vascular system *in planta*. This may be a mechanobiological remote signalling of the mechanical stress.

## Introduction

In nature the wind causes tree branches to bend transiently and repeatedly (Rodriguez *et al.*, 2008). Less transient bending may also occur when loads such as rainwater, snow, fruit, and sometimes the shoot itself weigh branches down (Cannell and Morgan, 1989; Alméras *et al.*, 2004). The common horticultural practice of artificially bending shoots of some species may have a positive qualitative and quantitative impact on flowering, fruit production, and maturation (Valinger, 1992; Kim *et al.*, 2004; Han *et al.*, 2007; Liu and Chang, 2011). The physiological and morphological consequences of bending are, however, not completely understood.

A rather generic syndrome of physiological responses to transient bending has been described and named thigmomorphogenesis (reviewed in Telewski, 2006; Coutand, 2010; Moulia *et al.*, 2011): branches and trunks tend to reduce their elongation (Coutand and Moulia, 2000) and to increase their radial growth (Coutand *et al.*, 2009), thereby reducing bending stresses in the shoot. In this complex physiological response, the plant senses the bending (mechanosensing) then a long-distance systemic signal moves acropetally from the stimulated tissue to the apical growth zone. The nature of this signal remains elusive (Coutand *et al.*, 2000; Moulia *et al.*, 2011). For a review of signalling of mechano-stimulation in plants, see Chehab *et al.* (2009). Long-term bending is also sensed by plants (Bastien *et al.*, 2013), leading to autotropic reactions that do not involve long-range signalling but rather proprioceptive sensing of the induced curvatures. Clear interspecific differences for these responses to bending were reported by Coutand *et al.* (2010) for short-term bending, and by Bastien *et al.* 2013 for long-term bending

The involvement of hormones in thigmomorphogenetic signalling was proposed by Neel and Harris (1971). However, this was readily questioned by Parkhurst *et al.* (1972) who suggested that the bending of plant stems may induce cavitation and conductance losses in the xylem, resulting in water stress and a decline in transpiration rates. A decrease in the conductivity of pine tree trunks was indeed observed shortly after trunks were bent by wind-sway, and this was related to damage induced in the xylem (Liu *et al.*, 2003). Many branch vessels proximal to branch attachments stop conducting water when branches sway in the wind. Vessels may also tear and become momentarily leaky at the point of branch attachment, causing cavitation (Tyree and Zimmerman, 2002). Reduced rates of net photosynthesis, transpiration, and stomatal conductance have been measured in rose shoots after they were bent (Kim *et al.*, 2004). Bending strains might therefore, either directly or indirectly, affect the integrity of the cross-sectional area of vessels, instantaneously decreasing the hydraulic conductance of stems. Thus the so-called thigmomorphogenetic reactions (e.g. decrease in primary elongation) may be partly confounded with a short-term water stress effect resulting from the physical effect of bending on hydraulic conductance. However, these direct effects of transient bending on hydraulic conductivity and safety have not been investigated and demonstrated.

The distance over which thigmomorphogenesis occurs (e.g. from the distal part of a branch several metres long to a growing meristem) implies that a long-range signal is involved. It is unlikely that classic signalling molecules (tRNA, hormones, etc.) could be transported rapidly enough in the xylem sap. Although the velocity of the signal has yet to be measured accurately, it is at least three times faster than any mass flow transport in the xylem sap (Moulia *et al.*, 2011). The possible involvement of an air-borne biochemical signal such as ethylene was also ruled out by Erner *et al.* (1980) in experiments on bean shoots. The acropetal long-distance signal may thus be physical in nature.

Electrical signalling is one form of physical signalling that has been considered. In many plant species, action potentials were only found to be transported over short distances in stems. A ‘slow electric wave’ was found to be transmitted in one aster species (Vian *et al.*, 1996), which might rely on subjacent changes in turgor pressure due to a pressure wave along the stem. Transient changes in water flow in the sap circulation system upon mechanical bending have thus been hypothesized as a mechanism for transmitting the long-distance signal in the thigmomorphogenetic growth response in primary growth zones (see the review by Moulia *et al.*, 2011). Bending causes a change in volume of the cells along the stem, and calculating the overall volume change that is incurred is currently the best way of approaching the subject of how a mechanosensitive signal might travel. When the stem is deformed, water expelled from the symplast and apoplast could lead to water movements and pressure variations in the xylem (Moulia *et al.*, 2011). Supporting this, Malone and Stanković (1991) reported that a series of swellings and shrinkages, strongly characteristic of a hydraulic pulse, were propagated along the stem after mechanical stimulation. However, this previous example only dealt with effects of drastic (and destructive) mechanical stress, so short-term hydraulic impacts of temporary and non-destructive mechanical bending remain unclear.

The first aim of this study was thus to analyse the impact of transient or steady mechanical perturbation on the conductivity and sap pressure levels in the xylem of detached bent shoots and to compare the response of gymnosperm and angiosperm species differing in wood density and cross-sectional anatomy. It was hypothesized (i) that transient bending may produce transient variations of flow, pressure, and conductivity in the xylem that could be the support of fast long-distance signalling along the stem, both in isolated stem segments and *in planta*; and (ii) that differences in anatomical structure between conifers and broadleaves could entail different hydraulic response to bending,

The behaviour of isolated stem segments was then compared with that of whole stems of an intact living plant. Water transport was also compared in the compression and tension sides of the bent shoot and in live and dead tissue.

## Materials and methods

### Plant material and experimental planning

Trees were grown in an orchard at the INRA site of Crouël, Clermont-Ferrand, France [N 45.8°, E 3.2°].

Bending experiments on fresh segments of single shoots of mature trees were conducted from April to May. Two angiosperms, *Carpinus betulus* L. and *Ilex aquifolium* L., and three gymnosperms, *Pinus sylvestris* L., *Cupressus sempervirens* L., and *Taxus baccata* L., with different characteristic wood densities were investigated. For brevity the species will be referred to by the genus only. Straight branches were carefully cut from the plant and brought to the lab. Branches were then cut into 40cm long segments that were used as specimens for mechanical and hydraulic measurements.

Bending was analysed *in planta* in 2-year-old poplar scions (*Populus alba×tremula* L. hybrids) that were grown in plastic containers (2 litres) and had attained 2 m in height. Experiments were conducted in the laboratory at room temperature in March.

### Four-point bending tests with flow monitoring

**Fig. 1.**
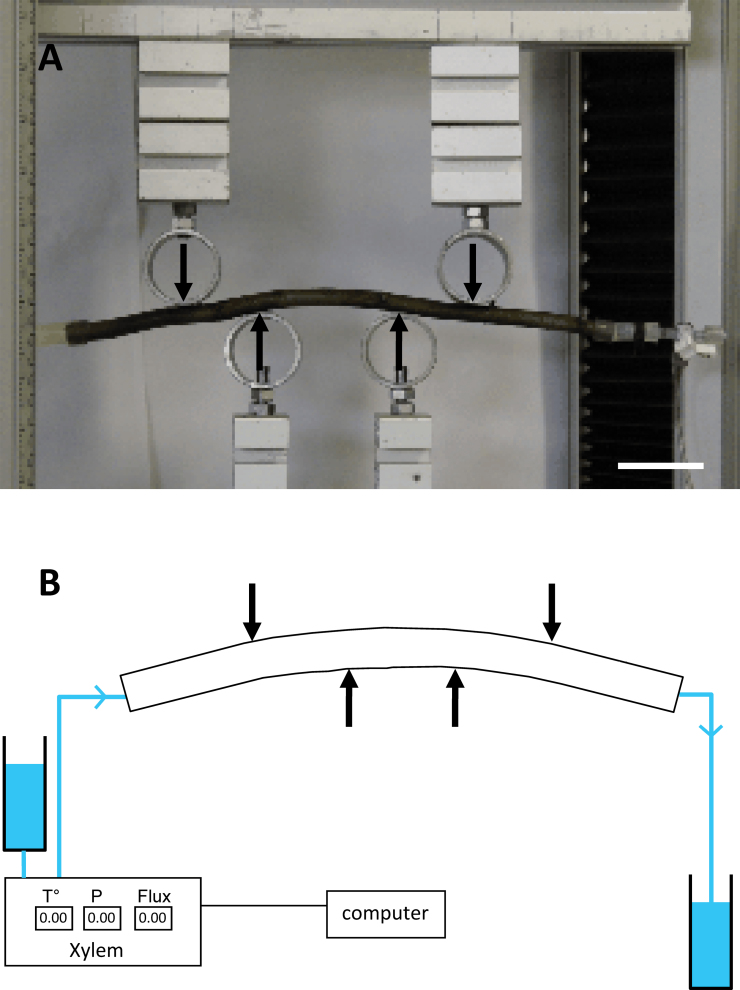
Photograph (A) and schema (B) of the experimental set-up for hydraulic pulse measurements. The extremities of the shoot are plugged into a XYL’EM system that generates the pressure differential and measures the hydraulic flow through the shoot while the strain is applied by the four-point bending device. In each bending step described in the Materials and methods, the upper cross-beam is lowered 5mm. (A) Scale bar=50mm. (B) Thick arrows indicate the bending points; thin arrows indicate the direction of water flow. (This figure is available in colour at *JXB* online.)

Five branches per species were bent with an Instron 5565 testing machine (Force cell 5kN) using a custom-designed four-point set-up (Fig. 1). A constant bending moment was applied in the central part of the specimen. Large diameter supports were used to enlarge the contact surface at the pressure points to limit the transversal local crushing of the sample through Hertz contacts. Bending point spacing was 160mm between the external supports and 80mm between the internal supports (Fig. 1). One end of the branch (apical part termed ‘upstream’) was plugged into a XYL’EM apparatus (Cochard *et al.*, 2002) which simultaneously measured water flow, the hydrostatic pressure gradient, and water temperature at a frequency of 1 Hz. The other end of the sample (basal part termed ‘downstream’) was connected to another tube to create a pressure differential that generated a flow of water that differed according to the hydraulic resistance of the branch. Branch or stem diameters were measured with an electronic caliper.

A standard bending experiment consisted of lowering the external loading points in four steps of 5mm each at 5mm s^–1^ to bend the sample further. After each displacement, the deformation was maintained for 60 s in order to record the steady response of water flow. Once the maximum flexion (20mm vertical displacement of pressure points) was reached, the external pressure points were raised in four 5mm unloading steps. The applied force and displacement were recorded at a frequency of 5 Hz. When the sample had steadied after each step, a photograph of the specimen’s curvature was recorded with a digital camera (Olympus SP570).

The total volume of water moved in each step was calculated as the integral of the variation in flow. To compare samples from species with large differences in initial water flow and conductivity, the initial measured flow value was subtracted from computed values.

### Killing live cells in branch segments

The role of living cells as a water reservoir was assessed using two branches of *Carpinus*. After measuring their initial hydraulic conductivity following the previous protocol, branch segments were placed in an autoclave at 120 °C and 200 kPa for 30min. This treatment causes cell lysis without dehydrating the sample (Améglio *et al.*, 2001). Branch segments were tested in the standard bending experiment.

### Estimation of volume of expelled water without initial flow

The volume of water expelled from a saturated sample during bending was measured by weighing. The two ends of a *Carpinus* branch (five samples) were connected to silicone tubes. The tubes were totally filled with water and their free ends were placed in a beaker filled with water installed on a precision balance. The sample was bent and released with the same Instron 5565 testing machine, and weight changes were recorded.

### Measurement of pressure pulses

The ends of two branches of *Carpinus* were equipped with pressure sensors (Honeywell 26PCDFA6D). The previous standard set-up (four loading then four unloading steps) was used to bend the branch samples with the testing machine while the pressure signal was recorded by a data-logger (Delta T SL1 6629) at a 1 Hz frequency.

### Comparison between compression and tension sides

Ends of samples were split horizontally so each longitudinal half of the stem could be separately connected to a XYL’EM apparatus to monitor the variations in the water flow of the tension (upper half) and compression (lower half) sides while bending the samples. At the end of the experiment, safranin and astra blue stains were injected, respectively, into each hydraulic system (stressed under compression or under tension) in order to dye the functional vessels. The branch was then cut into 20mm long slices to visualize the routes the water followed. This experiment was carried out with nine samples in total: five samples of *Carpinus*, two of *Ilex*, and two of *Cupressus*.

### Quantification of the mechanical strain states

For each step of the bending protocol, the maximum strain was computed according to beam theory applied to branches (Moulia and Fournier, 1997). Both the radius of the stem *r* and the radius of curvature *R* in the central part of the sample (between the internal pressure points) were measured. The curvature of the central part of the sample (between the internal contact points of the bending device) was evaluated by image analysis (ImageJ, Rasband, 2013) using the photographs of the sample. After segmentation, the coordinates of the points of the skeleton image were extracted and fitted with an arc equation, to give the mean radius of curvature *R*. Assuming that the branch segments were symmetric, the maximum longitudinal strain at the surface of the sample was defined as the ratio:

$$\varepsilon_{L\;\max}=\frac{r}{R}$$ (1)

### Estimation of wood basic density and lumen and cell wall volume

After the bending tests, 2.5cm long segments were cut out of the specimens. The green volume of the wood sample was determined according to Archimedes law. Samples were stored at 105 ºC for 48h then the dry weight was recorded. Basic density ρ_*i*_ was calculated as the ratio of dry weight to green volume.

The volume fraction contained within lumens and cell walls in the bent part of a branch can be estimated using the basic density and the sample volume. For this it can be assumed that (i) the fibre saturating point (FSP) is the water content when the free water contained in the lumen is removed; and (ii) the value of which is commonly accepted to be ~30% although a small interspecific variability may be observed (Vick, 1999). The mass (and volume) of water *M*_water cw_ that is in the saturated cell walls can therefore be evaluated as follows:

$$M_{\text{water cw}}=0.\text{3}V_{\text{S}}\;\rho_{i}$$ (2)

where ρ_*i*_ is the basic density and *V*_S_ is the volume of the sample (bent part only). In the same way, the volume of cell walls, *V*_CW_ in the sample can be roughly estimated using the cell wall density (*ρ*_0_=0.54g cm^–3^).

$$V_{CW}=\frac{M_{0}}{\rho_{0}}$$ (3)

Then, the total lumen volume can be estimated as:

$$V_{\text{lumen}}=V_{\text{sample}}–V_{\text{CW}}$$ (4)

Assuming the samples were fully saturated, the total mass of water *M*_water_ in the sample can be written as:

$$M_{water}=V_{lumen}\;\rho_{water}+M_{water_{CW}}$$ (5)

### Calculating the variation in the volume of water in the bent part of the sample due to bending strain

The change in volume of the sample due to bending can be expressed as:

$$\Delta V=\frac{1}{2}\underset{V_{S}}{∭}\varepsilon \;dV=\frac{1}{2}V\;\varepsilon_{Lmean}$$ (6)

where

$$\varepsilon_{Lmean}=\frac{2}{\pi }\;\varepsilon_{L\max}$$ (7)

According to Equation 6, variation in the volume of water contained within the cell wall can be computed as follows:

$$\Delta V_{water_{CW}}=\frac{1}{2}V_{water_{CW}}\;\varepsilon_{Lmean}$$ (8)

where *V*_water cw_ can be estimated according to Equation 3 and ε_Lmean_ according to Equations 1 and 7.

### *In planta* bending experiments

The pressure level in the vascular system of transpiring plants is strongly negative (Tyree and Zimmerman, 2002). For this reason it was not possible to place invasive probes into the vasculature to measure pressure. To circumvent this problem, a dormant scion that displayed physiological root pressure was used. It is possible to keep the xylem pressure positive in such a case by supply of nitrogen (in the form of nitrate) to generate physiological root pressure with full hydration of the aerial part (Ewers *et al.*, 2001; Delaire, 2005). Thus, the xylem pressure becomes positive. It was assumed that the relative pressure variations induced by bending are not pressure dependent and are representative of what could happen during transpiration.

The experiment was done in March, before poplar budburst. A 2-year-old poplar scion (height 2.0 m), growing in a plastic container (2 litres) filled with perlite substrate, was continuously perfused with a nutrient solution containing nitrate (1.82 mmol l^–1^ NO_3_^–^, 0.19 mmol l^–1^ H_2_PO_4_^–^, 0.24 mmol l^–1^ SO_4_^2–^, 1.00 mmol l^–1^ K^+^, 0.39 mmol l^–1^ Ca^2+^, 0.355 mmol l^–1^ Mg^2+^, and 0.2% Kanieltra^®^ micro elements). This treatment generated a high root pressure that increased the pressure in the vascular system of the stem. Two pressure sensors (Honeywell 26PCDFA6D) were plugged into the xylem through a specially designed needle similar to those used in Actinidia rootstock experiments (Clearwater *et al.*, 2007). The first needle was stuck into the xylem 10cm up from the stem collar and the second needle 90cm above the first. Pressure data were recorded with a data-logger (Omegasbus D5131). After the xylem pressure became positive (~40 kPa), the poplar trunk was rapidly bent around a rounded solid support, thus creating curvature of a known radius *R*. In order to achieve a steady strain rate, the bending was generated by dropping a 1kg mass attached to the upper part of the stem (Fig. 3). Three different levels of curvature were used. The maximum and mean longitudinal strain in the stem can be evaluated respectively as:

**Fig. 2.**
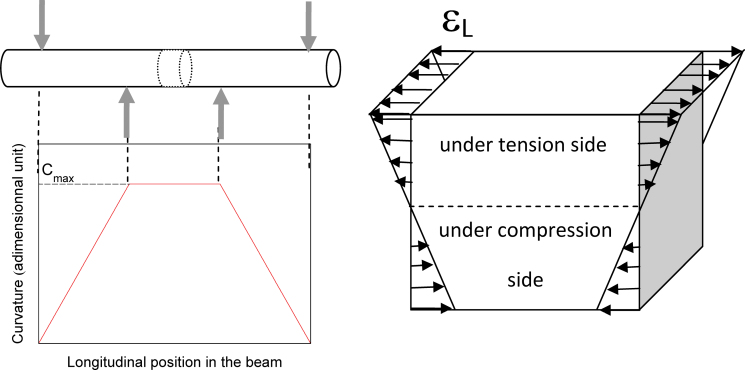
Theoretical profile of curvature along the beam and internal strain distribution in a beam section during a bending test. The longitudinal strain ε_L_ is maximum at the upper (positive values) and lower faces (negative values). Grey arrows indicate the positions of the four bending points. (This figure is available in colour at *JXB* online.)

**Fig. 3.**
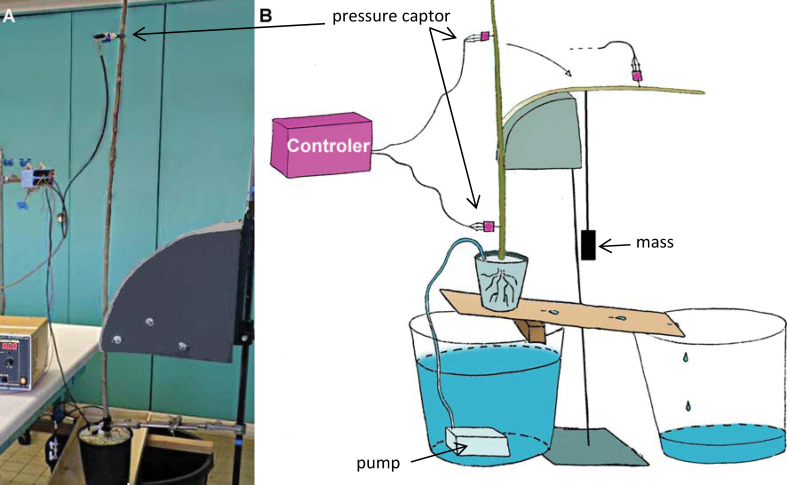
Experimental set-up for *in planta* bending and pressure measurements. The poplar tree (scion in dormancy) is continuously perfused with a nutrient solution containing nitrate applied with a small pump. This treatment generates a high physiological root pressure that refills xylem at the whole-plant level and increases the sap pressure in the vascular system of the stem so the pressure becomes positive. The stem was bent quickly by dropping a mass around a circular support with a known radius of curvature. Two sensors were placed at basal and apical positions of the stem to measure the xylem pressure. (This figure is available in colour at *JXB* online.)

$$\varepsilon_{L\max}=\frac{r}{r+R}$$ (9)

$$\varepsilon_{Lmean}=\frac{2}{\pi }\varepsilon_{L\max}$$ (10)

### Statistical analysis

To assess the effect of species on the volume of water expelled and the recovery time, general linear models were used to calculate repeated measures analyses of variance including the square radius of the sample as a covariate. Differences between levels of significant predictors were tested by Duncan’s multiple-range tests. To investigate the relationship between the hydraulic and mechanical properties, Pearson correlation coefficients were calculated. The analyses were performed with Statistica (StatSoft, Tulsa, OK, USA).

## Results

To investigate the mechanical effects of bending on plant shoots, two sets of experiments were conducted. The first set measured the impact of temporary or permanent mechanical bending on the conductivity and sap pressure levels in the xylem of detached shoot segments. The second set assessed the consequences of bending on xylem pressure *in planta.* The general working hypothesis was that transient bending may produce permanent or transient variations of flow, pressure, or conductivity in the xylem. Another consideration was whether samples from different species with different anatomical structure and hence physical characteristics would behave differently in response to bending.

### Water is expelled from xylem when stems bend, reducing water flow and generating a hydraulic pressure pulse

**Fig. 4.**
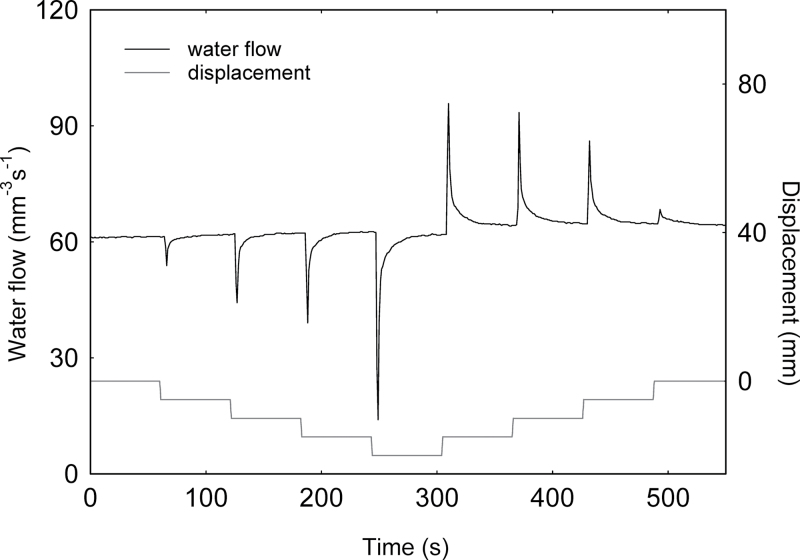
Typical pattern of water flow in a branch segment of *Carpinus betulus* subjected to four-point bending. The bending was applied in four mechanical steps with a vertical displacement of outer loading points of 5mm each at 5mm s^–1^ with an interval of 60 s between steps (grey curve). The initial position was recovered following the four inverse unloading steps at the same speed and intervals. Xylem water flow is shown by the black curve. During the bending of the branch, the water flow decreased rapidly for a few seconds then recovered its initial state.

**Fig. 5.**
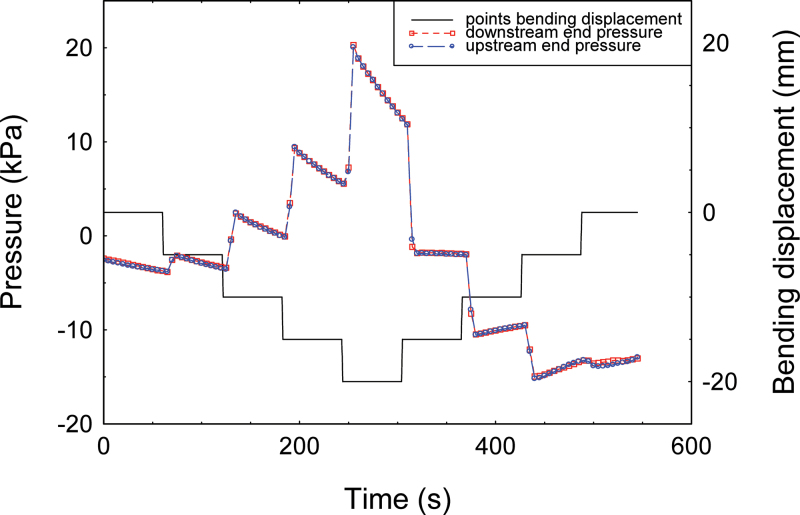
Pressure variations at the two ends of one branch segment of *Carpinus betulus* during a four-point bending test. When stem ends are sealed, bending generates a rapid pressure increase in the vascular system. Then small leaks in the system relax the pressure level. Unloading generates the opposite pressure variation from loading. (This figure is available in colour at *JXB* online.)

Bending of a branch segment caused a rapid and significant expulsion of water from the sample, which reduced the flow of water upstream. Figure 4 shows a typical example of flow recording. The decrease in flux was almost instantaneous, in terms of the 1 Hz sampling frequency. The magnitude of this change was such that sometimes water flow stopped completely. This initial rapid decrease in flow was followed by a progressive exponential recovery of flow back to the initial rate. The recovery time, computed as the half-life of the signal, was ~4 s (Table 1). Both the magnitude of the trough inflow (for simplicity considered a negative peak) and the recovery time increased in a non-linear way with an increase in branch curvature (Table 1). A symmetrically opposite signal was observed during unloading, with transient increases in upstream flow at each unloading step (Fig. 4). This hydraulic behaviour was generic and observed for all the tested species. The cross-sectional area of the sample was found significantly to affect how much water was expelled (Supplementary Table S1 available at *JXB* online), but not the recovery time. When both ends of a branch sample were sealed to create a closed vascular system, branch bending resulted in a large increase in pressure in the xylem (Fig. 5). Again, the magnitude of the change in pressure was greater when the applied strain was greater (data not shown).

**Table 1.** Interspecific variability of hydraulic pulse signals generated in samples from five tree species in four-point bending experimentsSpecies, referred to by genus only, are *Carpinus betulus* L., *Ilex aquifolium* L., *Pinus sylvestris* L., *Cupressus sempervirens* L., and *Taxus baccata* L. The bending was applied in four mechanical steps with vertical displacement of upper pressure points of 5mm each at 5mm s^–1^ with intervals of 60 s between steps. The magnitude of the signal refers to the maximum peak. Recovery time refers to the half-time of recovery of the signal, and volume refers to the total amount of water that is expelled from the branch.

Displacement (mm)	*Carpinus*	*Ilex*	*Pinus*	*Cupressus*	*Taxus*
	Magnitude of the signal (mm^3^ s^–1^)				
5	16.8±4.4	17.5±4.4	9.8±5.0	3.9±4.4	2.4±3.7
10	30.3±4.7	32.8±4.7	21.5±5.4	14.3±4.7	14.9±5.0
15	40.7±5.6	33.3±5.7	21.2±6.5	31.0±5.6	25.8±6.0
20	57.1±8.3	35.4±8.3	23.1±9.5	55.0±8.3	30.0±8.9
	Recovery time (s)				
5	4.7±1.1	3.2±1.1	6.8±1.2	7.1±1.1	2.8±1.1
10	4.8±1.1	4.3±1.1	7.0±1.2	8.4±1.1	3.6±1.1
15	5.3±1.4	5.2±1.4	8.5±1.6	8.1±1.4	3.9±1.5
20	6.6±1.2	4.8±1.2	13.2±1.3	10.1±1.2	5.4±1.2
	Volume (mm^3^)				
5	75.9±17.9	58.0±18	42.9±20.6	27.7±17.9	2.5±1.1
10	159.2±27.3	130.8±27.5	110.3±22.4	108.7±27.3	47.0±9.2
15	203.6±38.8	169.9±39.2	140.5±34.6	251.8±38.8	82.3±21.0
20	365.3±66.8	188.4±47.4	269.7±66.8	521.1±66.7	138.4±31.5
	Basic density (g cm^–3^)				
	0.54±0.01	0.53±0.1	0.28±0.1	0.41±0.1	0.54±0.1

Values are mean±SD of five samples per species.

As a control to verify that the flow itself was not responsible for the expulsion of water, fully saturated samples were set up with no pressure difference between the two ends so there was no initial flow (no pressure difference between the two ends). Water was again expelled when the sample was bent and absorbed during unloading (data not shown).

### Wood density influences the rate of recovery from bending

Similar hydraulic behaviour was observed in branches from angiosperms *Carpinus* (hornbeam) and *Ilex* (holly) and gymnosperms *Pinus* (Scots pine), *Cupressus* (Mediterranean cypress), and *Taxus* (European yew), even though the xylem structures of these five species differed. Statistically significant differences in the recovery times (*F*-value=3.83, *P*<0.05) and in the total volume of water expelled (*F*-value=3.84, *P*<0.05) were observed (Table 1; Supplementary Table S1 at *JXB* online) between different species. More water was expelled during bending from *Carpinus* and *Cupressus* samples than from *Pinus* and *Ilex*, while *Taxus* expelled the least. The basic densities of *Carpinus*, *Ilex*, and *Taxus* are quite similar so there is no clear relationship between the volume of water expelled and the basic density of the wood. In contrast, samples from these three species with the highest basic densities recovered their initial state more quickly than the lower density species *Pinus* and *Cupressus*.

Live cells in *Carpinus* branch samples were killed by autoclaving, but this did not greatly alter the hydraulic response profile. The only differences detected were that slightly more water was expelled (+5%) and the recovery time was slightly longer for the autoclaved samples.

### Compression versus tension effects on the hydraulic signal

According to beam theory, the convex outer half of the bent beam is under longitudinal tension while the concave inner half is under longitudinal compression (Fig. 2). In between is the so-called neutral line, where strain is null. In the experimental set-up used here (Fig. 1), the upper half of the sample was under tension while the lower part was under compression. The ends of the sample were split horizontally so the water flow through each half of the stem could be monitored separately while bending the samples as before.

**Fig. 6.**
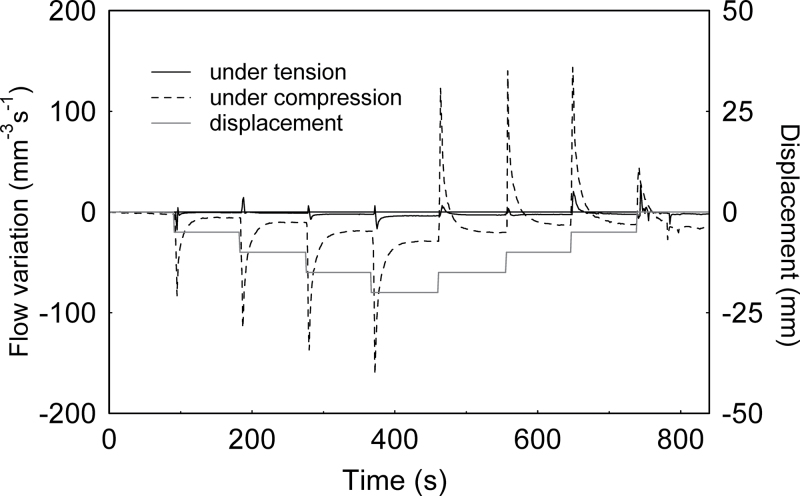
Comparison of the flow patterns along the side under tension and the side under compression of a branch of *Carpinus betulus* during bending tests. Initial values of water flow are subtracted from the measured values.

**Fig. 7.**
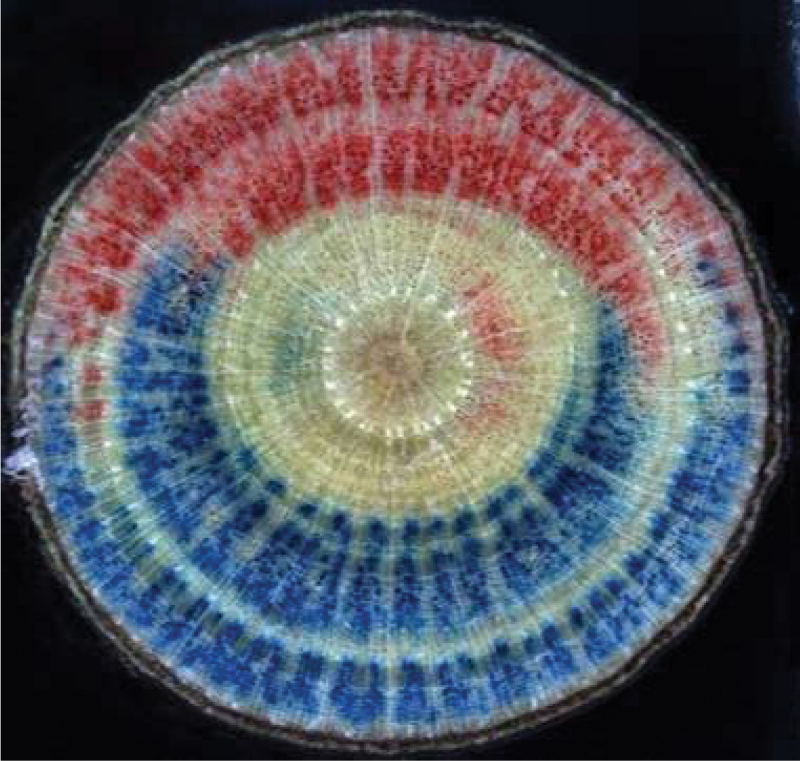
Hydraulic functional tissues. After mechanical bending, the upper longitudinal half of the sample that experienced tension strain was perfused with red safranin (top) and the lower half that experienced compression strain was perfused with astra blue (bottom) in order to stain the hydraulic pathways. Staining confirmed that the grain angle of the wood was negligible and that water flowed longitudinally from end to end (*Carpinus betulus*). (This figure is available in colour at *JXB* online.)

The side of the branch under compression (lower side on Fig 1) generated larger hydraulic effects than the part under tension (upper side). While the side under tension showed a slight positive peak for the first step followed by slight negative peaks, the compressed side alone produced almost the whole profile that was obtained with shoots that had not been split at the ends (Fig. 6). Split-dye experiments confirmed that water transport occurred along one side of the branch or the other. The red and blue dyes did not mix, showing that dye was not readily transferred between the compression side and the tension side and that water mostly flowed longitudinally (Fig. 7).

### Relationship between deformed volume and volume of water expelled in the hydraulic pulse

**Fig. 8.**
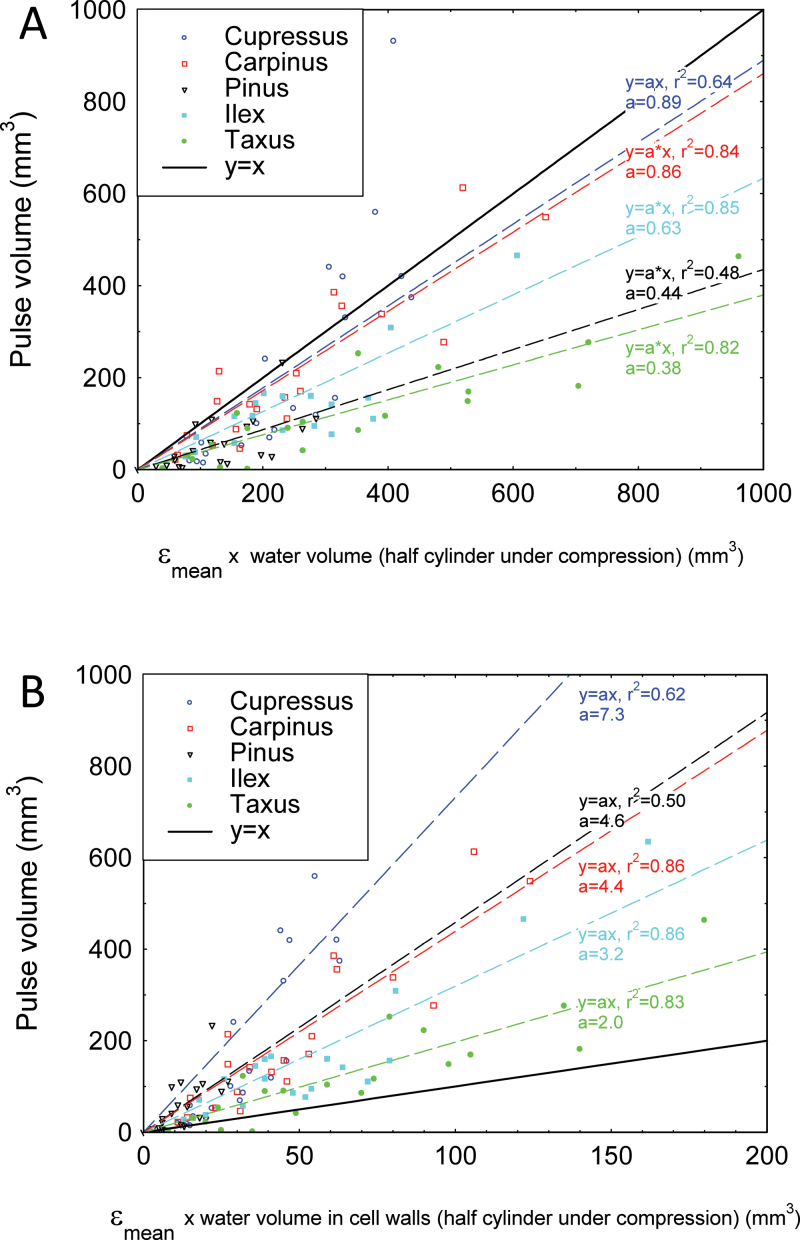
Comparison of hydraulic pulse magnitude in different tree species. The relationship between the mean longitudinal strain ε_mean_×initial water volume and the water volume expelled from the shoot during bending was plotted. The variation in the volume due to bending was computed for either (A) all the compartments, that is lumens and cell walls, or (B) cell walls only according to Equations 6 and 8, respectively (see the Materials and methods). (This figure is available in colour at *JXB* online.)

When a slender rod like a branch or stem is bent, there is a concomitant change in volume. Equations 3–5 were used to predict the change in the volume of water contained in the bent part of the sample when bending strain is applied. Calculated values were compared with the measured volume of water expelled in the hydraulic pulse. Figure 8A shows that the volume of expelled water was positively correlated with the calculated change in volume of water due to strain, ranging proportionally from 40% to 90%. The volume of expelled water was, however, 2–8 times larger than the possible change in the volume of water contained in the cell walls alone (Fig. 8B). This shows that the expelled water probably came from both the lumen and the cell wall. Large differences were observed between samples from different species, but no correlation could be made with the plant group (angiosperm or gymnosperm) or to wood density.

### *In planta* bending experiments

Xylem pressure was measured at upper and lower positions, 0.9 m apart (bending occurred in the centre), of stems of poplar hybrids. Although the pressure in the vascular system of transpiring plants is usually strongly negative (Tyree and Zimmerman, 2002), precluding its measurement, physiological conditions were manipulated so that a positive stem pressure developed (see the Materials and methods). Initially, the pressure was positive and equal to 47 kPa and 38 kPa, respectively, in the lower and upper parts of the stem. The pressure difference ΔP (9 kPa) corresponded exactly to the hydrostatic pressure of the 0.9 m high water column Δh, calculated as ΔP=ρ_water_ g Δh.

**Fig. 9.**
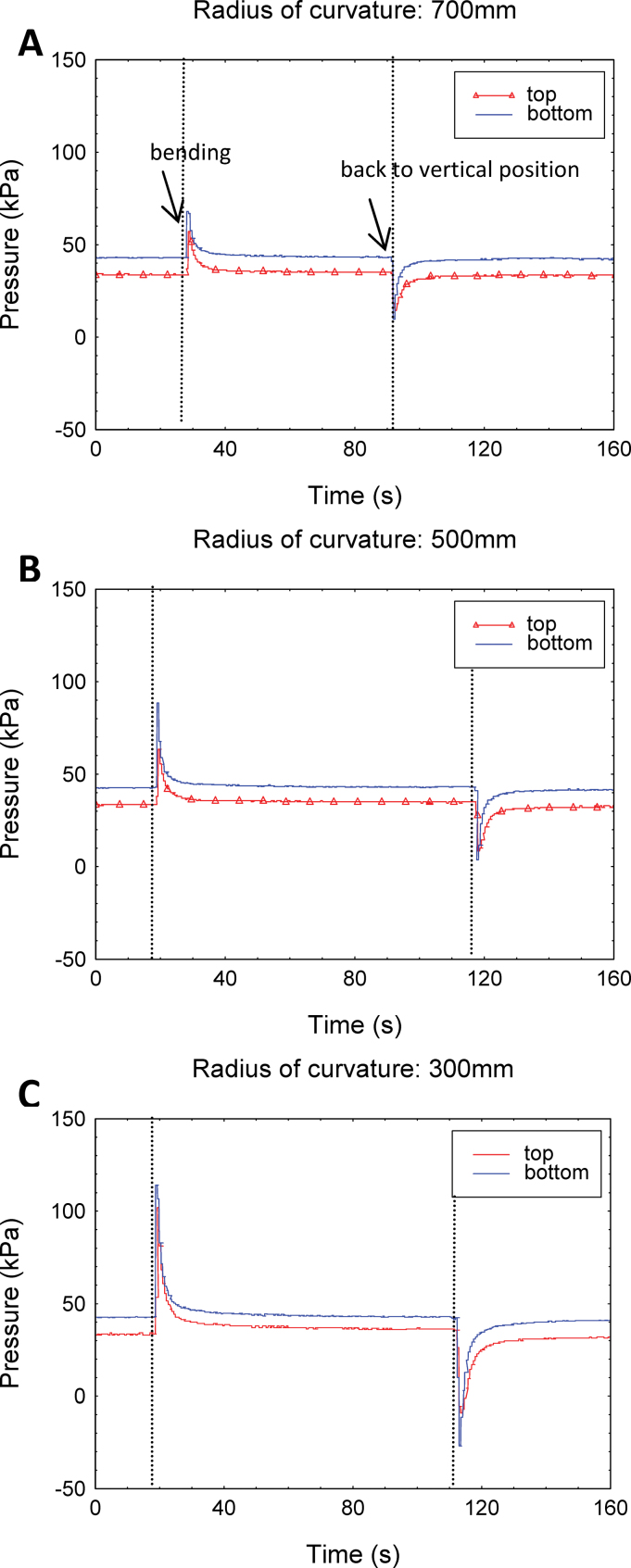
*In planta* hydraulic pressure pulses. Pressure variations in the upper and lower regions of poplar stems (*Populus alba×tremula* L. hybrid) when bending occurs in between those two regions. The pressure increase was very fast and the recovery lasted a few seconds. The magnitude of the signal increased with the magnitude of the mechanical strain, that is inversely to the curvature. Radius of curvature was 700mm (A), 500mm (B), and 300mm (C). Bending speed was constant. Permanent differences between upper and lower pressure corresponded to the hydrostatic pressure of the water column between the two sensors 0.9 m apart. (This figure is available in colour at *JXB* online.)

**Fig. 10.**
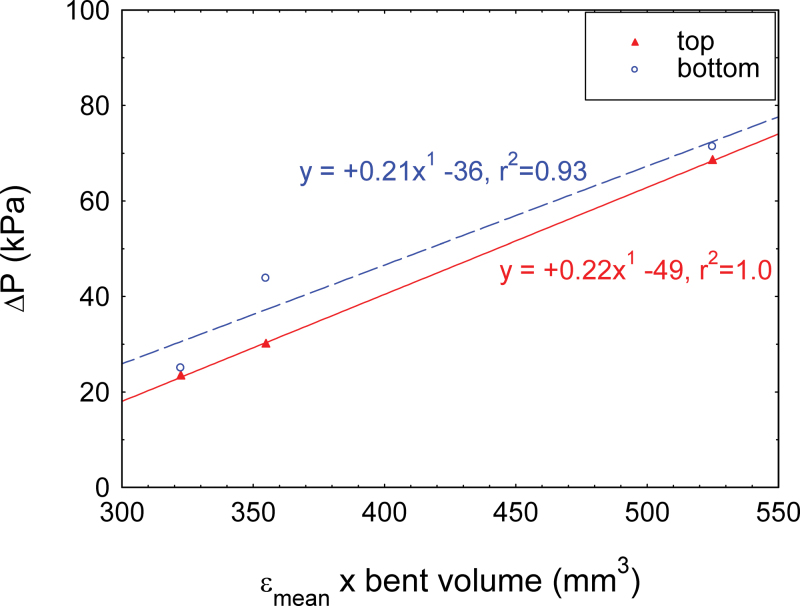
*In planta* hydraulic pressure pulses. The relationship between the magnitude of the hydraulic pressure pulses and the mean applied longitudinal strain ε_mean_ (see Equation 3) is plotted. Bent regions were 48, 54, and 63cm long, respectively, for ε_Lmax_ of 1.8, 1.09, and 0.85%. The total distance between the lower and upper sensors was 0.9 m. (This figure is available in colour at *JXB* online.)

Once the xylem pressure became positive, the poplar stem was rapidly bent around a curved support by dropping a 1kg mass attached to the upper part of the stem (Fig. 3). Three different supports were used with different radii of curvature, 300, 500, and 700mm. Rapid bending generated transient hydraulic pressure peaks in both pressure sensors (Fig. 9). The maximum increase in pressure was 70 kPa when the radius of curvature was 300mm, that is when the applied strain was 1.8%. After a relaxation time lasting several seconds, a new steady state was reached. At both sensor positions the final steady pressure was slightly higher than the initial one. Variations were symmetrical on either side of the bent region. The pressure difference between upper and lower sensors decreased compared with the initial state. This is because the water column Δh is shorter after bending. The magnitude of the transient pressure peaks was largely dependent on the magnitude of the applied strain (Fig. 10).

**Fig. 11.**
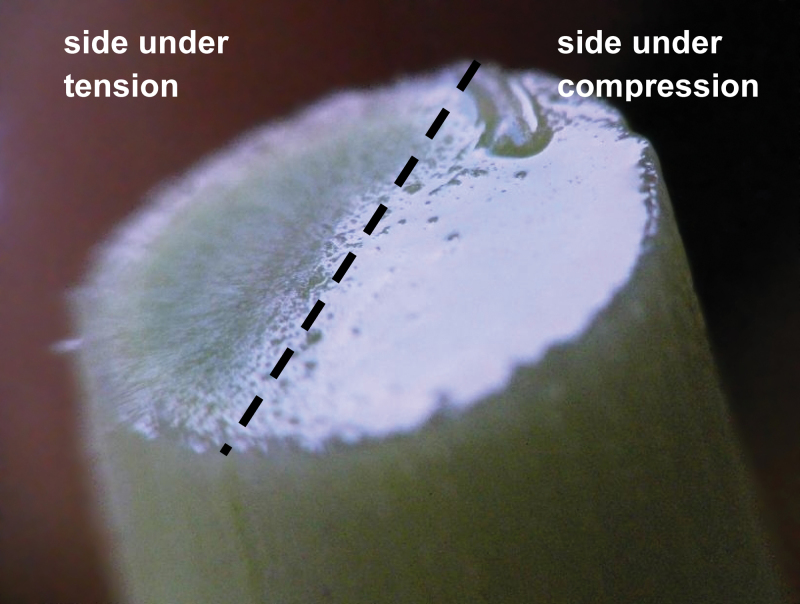
Expulsion of water at the end of a bent branch. Water is only expelled from the xylem on the compressed side (image from Supplementary Video S1 at *JXB* online). (This figure is available in colour at *JXB* online.)

When the stems returned to the upright position, a pulse of low pressure was detected at both positions before pressure returned again to a slightly lower pressure steady state.

## Discussion

### Mechanical strain does not affect conductivity

Previous studies suggested that bending might impair water conductance through air embolism or vessel crushing (Spicer and Gartner, 2002; Kim *et al.*, 2004). It was found here that the mechanical bending of a branch never induced a steady change in the water flow, that is in the conductivity of the hydraulic system. Variations in flow occurred during loading or unloading but were transient (a few seconds) and reversible (Fig. 4). The hydraulic conductive system recovered its initial efficiency even beyond the elastic zone of the wood, except for the largest deformations close to the mechanical break point (in which case the conductance only slightly decreased; data not shown). No effect of short-term bending on conductivity behaviour was observed, and this was generic to all the species studied. This shows that the transverse deformation of the vascular structure (due to Poisson ratios during bending) was not sufficient to modify the hydraulic behaviour (Cochard *et al.*, 2004). The hypothesis that sustained bending has an impact on conductivity should thus be rejected. Consequently, the fact that some hydraulic properties were modified when a branch was permanently bent, as reported by Spicer and Gartner (2002), is probably a long-term effect due to the formation of new cells with special hydraulic properties, for example reaction wood or as a result of plastic mechanical damage to the existing conductive pathway as suggested by the concurrent changes in the Young’s modulus and the rupture modulus (Fredericksen *et al.*, 1994).

### Mechanical strain induces a hydraulic pulse

Here a novel phenomenon was measured, the onset of a transient hydraulic pulse upon mechanical bending. This pulse was revealed through dual experiments that monitored (i) flow across the ‘open’ xylem or (ii) pressure in sealed xylem. Both types of experiment showed that deformation of the shoot induced the expulsion of water from the xylem, coinciding with a peak in sap pressure in the places where this outflow occurs. Water expulsion from the xylem during bending was even visualized directly (Fig. 11; see Supplementary Video S1 at *JXB* online). This validates the hypothesis that transient bending produces hydraulic pulses in the xylem both in isolated stem segments and *in planta*. Moreover, the hydraulic pressure in the xylem and the volume of the flow pulse increase proportionally to the magnitude of the applied mechanical strain (Fig. 10). The volume of water that is involved in the signal is consistent with the variations in volume of water reservoirs in the bent part of stems.

### Mechanism of strain-generated hydraulic pulses

The reservoir for this strain-expelled water is not the living cells, as dead stems did not have a significantly different pressure signal from live stems. The hydraulic pulse reported here is hence related solely to the deformation of the apoplast. This behaviour calls to mind the poroelastic properties of the vascular system, a double porosity structure. Cell lumens have a large porosity and cell walls a very small porosity (Kameo *et al.*, 2009; Nguyen *et al.*, 2010).

Assuming that water-saturated wood material performs like a poroelastic spongy material (Bader *et al.*, 2011), the process of strain-induced pressurization and water expulsion could be modelled as follows. For simplicity, just the side under compression during bending can be considered, where the volume of lumens and cell walls decreases mainly according to the longitudinal strain ε_L_ and water is expelled. The pressure rapidly increases locally and this energy is propagated along the lumen conduits like a hydraulic pulse. Two cases need to be considered. (i) If the system is open, as in the first experimental set-up where flow was measured, the hydraulic pulse can be propagated, probably in large xylem vessels, carrying the amount of water that will be expelled from the sample. Once the pulse has passed, the flow returns to its initial rate. (ii) If both ends of the hydraulic system are closed, the local pressure increases in the entire sample according to the magnitude of the strain.

When the water flow in the tension and compression sides of bent branches were compared separately, large differences were observed. The side under compression showed large decreases in water flow when loading and slow recovery of flow when strain was unloaded. Effects on flow through the side under tension were much smaller. Since no transverse flow of water was observed from one side to another in dye experiments, tension and compression thus have non-symmetrical effects. Such non-symmetrical behaviour between compression and tension sides cannot be explained by classical elastic beam theory and probably involves non-linear physical behaviour that remains to be elucidated.

### A generic phenomenon with high interspecific variability

Hardwoods *Carpinus* and *Ilex*, and softwoods *Pinus*, *Cupressus*, and *Taxus* have a wide range of xylem conductivity, and contrasted anatomical structures and wood density. Strain-induced hydraulic pressure pulses were observed in both hardwoods and softwoods. However, the ratio between the volume of water expelled during a pulse and the mean longitudinal strain varies from 1 to 2 among species.

It may be hypothesized that these differences are related to differences in wood density. Assuming that the moisture content in the cell wall is always ~30% and that the dry mass density ρ_0_ of the cell walls does not differ between conifers and angiosperms (Hacke *et al.*, 2001), the volume of water contained in the cell wall can be estimated as a function of the basic density from Equations 4–6:

$$\frac{V_{water_{CW}}}{V_{water_{Lumen}}}\approx 0.3\frac{\rho_{i}\;\rho_{0}}{\rho_{0}-\rho_{i}}$$ (11)

The volume of water in the cell wall represents 11% of the volume of water contained in the lumens for *Pinus*, 17% for *Cupressus*, and 24% for *Carpinus*, *Ilex*, and *Taxus.* This variability in the volume of water expelled from the cell wall does not explain all the variability in the signal magnitude observed among species (Fig. 8B).

### Relevance of strain-induced hydraulic pulse in transpiring plants

A crucial point to be discussed is whether the results obtained here when the xylem was under positive pressure (both in branch segments and in stems *in planta*) are relevant to the real situation of transpiring plants. Plants rarely experience positive root pressure naturally. According to the tension–cohesion theory, the water in the xylem is under negative pressure as soon as the leaves begin to transpire. Two lines of reasoning can be followed. (i) From a theoretical point of view, the water column in the vessel lumen is continuous. Any compressive strain induces a local decrease in tension in the column and modifies the pressure gradient in the xylem vessels in the same way, whatever the initial pressure whether positive or negative. (ii) From an experimental point of view, the pressure variations ΔP observed were exactly the same whatever the initial pressure (data not shown). According to these arguments, these results can be reasonably extrapolated to initial negative pressures and it can be hypothesized that mechanical strains generate absolute pressure variation ΔP, even in transpiring plants.

### Pressure pulse propagation as a candidate for long-distance mechanosensing signal transmission

Pressure variations transported along the vascular system of plants have been found to generate rapid physiological responses (Malone and Stanković, 1991;Tyree and Zimmerman, 2002). However, their exact pathway (xylem and/or phloem) has remained unknown and this phenomenon has only been described in response to wounding stresses such as localized burning (Malone and Stanković, 1991). It is suggested that the poroelastic pulse reported in this study is a good candidate for the rapid long-distance signalling of mechanical stress in plants (Malone, 1996; Coutand *et al.*, 2000). Since this hydraulic pulse is transported throughout the xylem, it could have a secondary impact on the turgor pressure of living cells, leading to cell deformation and physiological and molecular responses (Ortega, 2010).

Another line of support for the poroelastic hydraulic pulse as a candidate for long-distance signalling comes from the analysis of the thigmomorphogenetic responses to stem bending. The bending of the basal part of a stem generates a fast long-distance signal in the plant, affecting the apical growth zone (Coutand *et al.*, 2000; Pruyn *et al.*, 2000) even in grafted plants (Erner *et al.*, 1980). Significantly this signal has been shown to be proportional to the strained volume of the stem (Coutand and Moulia, 2000; Moulia *et al.*, 2011) and is the basis of the Sum of Strain-Sensing Model (S^3^m), that has been validated for both growth responses and mechanosensitive gene activation (Coutand and Moulia, 2000; Coutand *et al.*, 2009; Moulia *et al.*, 2011). The present results are fully consistent with the S^3^m model. The hydraulic pulse is highly correlated to the strained volume (Fig. 8), and is thus a quantitative long-distance signal that may be involved in thigmomorphogenesis (whose nature has remained elusive so far; see Chehab *et al.*, 2009 for a review of signalling of mechano-stimulation in plants). Moreover the *in planta* experiments carried out in this study proved that the hydraulic wave propagates along the vascular architecture symmetrically up and down. Such a signal could therefore induce a thigmomorphogenetic response in the root system when aerial regions of a plant are deformed by bending (Pruyn *et al.*, 2000; Kern *et al.*, 2005).

Finally, this same hydraulic mechanism could potentially provide an effective way of transmitting water status information over the relatively long distances of a tree’s structure (Malone, 1996). Soil water stress elicits a hydraulic response in the shoots of various plants faster than abscisic acid, the hormone involved in signalling abiotic stress in plants (Christmann *et al.*, 2007). Plant cell water channels, aquaporins, are known to be able to respond to variations in pressure (Wan *et al.*, 2004). Nevertheless, the mechanisms involved in the perception of the hydraulic signal by living cells and the transduction from a physical to a chemical signal that triggers acclimation responses remain to be elucidated (Christmann *et al.*, 2013).

## Conclusion

According to the experimental results obtained here, the hypothesis that bending has an impact on the hydraulic conductivity of branches or stems was rejected, even for large and sustained deformations. Long-term effects reported in the literature were possibly due to the formation of new reaction wood in the branches. Nevertheless, a new physical phenomenon in trees was discovered and measured. Transient bending of shoots generates a rapid and transient flow of water, which induces a pressure pulse that is able to propagate along the vascular system. Elastic beam theory cannot explain this behaviour. The origin of this hydraulic pulse is probably a form of non-linear poroelastic behaviour of the wood cellular structure. Investigations are now focusing on the possible mechanisms using microfluidic synthetic beams that mimic natural branches.

This strain-induced hydraulic pulse may be the postulated rapid long-distance signal of plant mechanical stress when branches and stems bend rapidly such as in the wind. Hydraulic pulses were propagated along the vascular system of the xylem to both the upper and lower regions of the stem. As a signalling process, this could be an efficient way to transport mechanical information to the extreme organs such as leaves, roots, and apices. The physiological or molecular responses to this transient pressure increase can now be investigated.

## Supplementary data

Supplementary data are available at *JXB* online.

Table S1. *F*-values for repeated measures analysis of variance of the magnitude of the signal, half-time of recovery, volume of water involved in each step of the bending process, and maximum longitudinal strain. ns, not significant, *P<0.05, **P<0.01, ***P<0.001.

Video S1. Expulsion and re-absorption of water at the extremity of a branch (*Carpinus betulus* L.) during bending and during the return to the upright position, respectively. Water appears on the side of the xylem that is under compression, starting in the most strained region.

Supplementary Data
