# Supplementary material for: Tree shoot bending generates hydraulic pressure pulses: a new long-distance signal?
Source: J Exp Bot. 2014 Feb 20;65(8):1997–2008. doi: 10.1093/jxb/eru045 (PMC3991735; doi:10.1093/jxb/eru045)
Supplement: Supplementary Data [file supp_65_8_1997__index.html]

Tree shoot bending generates hydraulic pressure pulses: a new long-distance signal? — Tree shoot bending generates hydraulic pressure pulses: a new long-distance signal? — Supplementary Data 

# Tree shoot bending generates hydraulic pressure pulses: a new long-distance signal?

## Supplementary Data

Data files

**Files in this Data Supplement:**

- Supplementary Data - Supplementary Data
- Supplementary Data - Supplementary Data
