# Supplementary material for: Tree shoot bending generates hydraulic pressure pulses: a new long-distance signal?
Source: J Exp Bot. 2014 Feb 20;65(8):1997–2008. doi: 10.1093/jxb/eru045 (PMC3991735; doi:10.1093/jxb/eru045)
Supplement: Supplementary Data [file supp_eru045_jexbot107284_file002.pdf]

Tree shoot bending generates hydraulic pressure pulses: a new long distance signal?

Rosana Lopez, Eric Badel, Sebastien Peraudeau, Nathalie Leblanc-Fournier, Francois Beaujard, Jean-Louis Julien, Hervé Cochard, and Bruno Moulia

SUPPLEMENTARY TABLE:

Supplementary Table S1. F-values for repeated measures analysis of variance of the magnitude of the signal, half-time of recovery, volume of water involved in each step of the bending process and maximum longitudinal strain. ns, not significant, **P* < 0.05, ** *P* < 0.01, *** *P* <0.001.

**Magnitude of the signal**		**Recovery time**		**Volume**	
Between-subjects effects		Between-subjects effects		Between-subjects effects	
Species	2.64 ns	Species	3.83 *	Species	3.84 *
Radius^2	20.08 ***	Radius^2	1.17 ns	Radius^2	21.42 ***
Within-subjects effects		Within-subjects effects		Within-subjects effects	
Displacement	3.04 *	Displacement	0.30 ns	Displacement	0.16 ns
Displacement *species	3.21 **	Displacement *species	2.21 *	Displacement *species	4.53 ***
Displacement * Radius^2	1.90 ns	Displacement * Radius^2	2.65 ns	Displacement * Radius^2	6.13 **
